# Relationship between patient satisfaction with medical doctors and the use of traditional Korean medicine in Korea

**DOI:** 10.1186/s12906-015-0879-x

**Published:** 2015-10-14

**Authors:** Dongsu Kim, Byungmook Lim, Changhee Kim

**Affiliations:** Policy Division, Korea Institute of Oriental Medicine, Daejeon, South Korea; Division of Humanities and Social Medicine, School of Korean Medicine, Pusan National University, Yangsan, South Korea; Division of Longevity and Biofunctional Medicine, School of Korean Medicine, Pusan National University, Yangsan, South Korea; #316 School of Korean Medicine, Pusan National University, Gyeongnam, 626-770 South Korea

**Keywords:** Traditional Korean medicine, Doctor satisfaction, Anderson behavior model, Korea health panel, Western medicine

## Abstract

**Background:**

Satisfaction with medical doctor (MD) has been studied as a possible motivation for trying complementary medicine. This study aimed to explore the relationship between Korean outpatients’ satisfaction with their MDs and their use of traditional Korean medicine (KM).

**Methods:**

Data were drawn from the 2011 annual Korea Health Panel, a national representative sample. We analyzed the relationship between outpatients’ use of KM and outpatients’ satisfaction with MDs by using the responses of 9,753 outpatients, including 1,946 KM outpatients. The Andersen behavior model was applied to select the variables. The validity and reliability of the questionnaires were tested by Factor Analysis and Cronbach’s alpha. Multiple logistic regression was used to evaluate five MD satisfaction indicators (patient’s trust in MD, MD’s careful listening, MD’s sufficient explanation, MD’s consultation time, and MD’s respect for patient) and the overall satisfaction with the MD.

**Results:**

There was no significant difference between the MD satisfaction of KM users and that of nonusers in any of the 5 indicators of MD satisfaction. When we controlled for all independent variables from the Anderson behavior model, however, the patients’ overall dissatisfaction with MDs was associated with their use of KM (OR = 0.87,0.76–0.99). In addition, the more a patient was dissatisfied with the consultation time of their MD, the more they used KM (OR = 0.82, 072–0.94).

**Conclusions:**

Patients who were dissatisfied with their MD were more likely to use KM; the main indicator affecting MD dissatisfaction was the relatively short time of MD consultations. This could be one reason why KM plays a complementary role with conventional medicine in Korea.

## Background

Recently, interest in and use of complementary and alternative medicine (CAM) has increased worldwide [1–4]. With this heightened social interest, many studies have been conducted to determine who uses CAM, why they use it and how. A previous study revealed that CAM users are younger, more active, thinner, and more educated and earn more than non-CAM users [5]. Moreover, the use of CAM has increased due to the growth in the number of patients with chronic diseases [3, 4], its reduced side effects relative to western medicine (WM), and the expansion of insurance coverage for CAM [2].

Another important factor that influences CAM use is the health beliefs of patients and the general public. Among many aspects of health beliefs, we focused on satisfaction with MDs, which has been studied as a possible motivation for trying CAM [6]. According to previous studies of CAM users’ beliefs pertaining to health care, CAM users have a holistic and independent health philosophy [7, 8], have incomplete trust in MDs [9], and have needs that have not been met by conventional medicine [6, 9–11]. However, previous studies have had limitations, such as not adjusting for the patient’s disease [9], region [6, 11], and gender [10] and being too specific to represent the overall population and sociodemographic characteristics [7].

To investigate the influence of MD satisfaction on the use of CAM, we conducted a cross-sectional analysis with Korea Health Panel data surveying a national sample of the Korean population on the use and cost of health care services. Among the various CAM modalities, we focused on traditional Korean medicine (KM), which is a representative modality of CAM in Korea and has a competing relationship with WM in some areas [12, 13]. We identified people’s characteristics and the factors of MD satisfaction associated with the use of KM.

## Methods

### Data source

We analyzed 2011 Korea Health Panel data collected jointly by the Korea Institute for Health and Social Affairs (KIHASA) and the Korean National Health Insurance Service. A Korea Health Panel sample was selected from the 2005 Korean Population and Housing Census data using region stratification variables. The data were initially collected from 7,009 households and 21,283 individuals in 2008, with 5,741 households and 17,035 individuals remaining in 2011. A detailed description of the Korea Health Panel was presented elsewhere [14, 15]. This study used public data from the Korea Health Panel that did not include any personal identification, and the survey conformed to local legislation and the Declaration of Helsinki. The data were provided by KIHASA with permission to use and analyze them.

### Sample selection

**Fig. 1:**
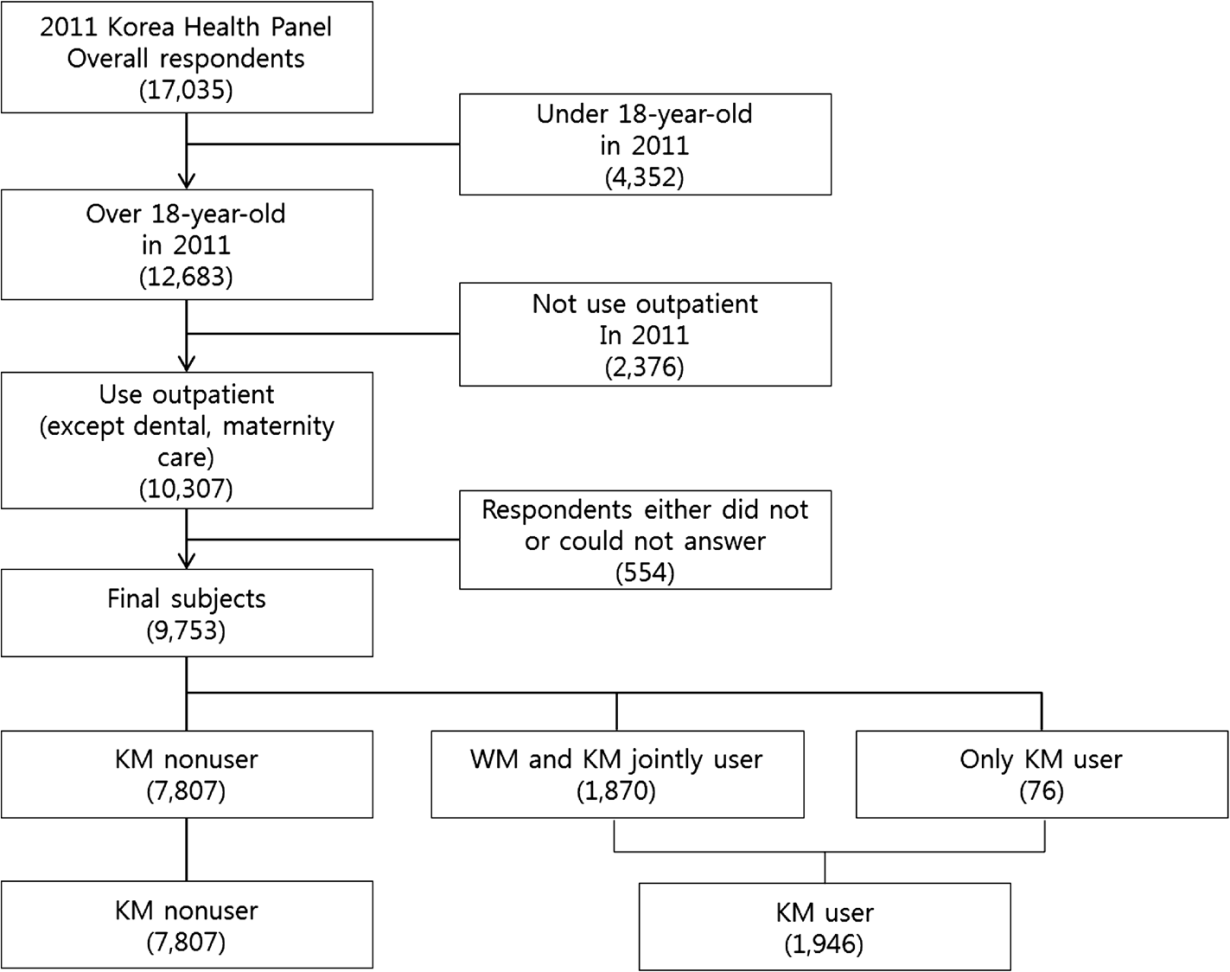
Selection process of study subjects

We selected and analyzed individuals over 18 years old who used outpatient services in 2011. A total of 4,352 individuals under 18 years old in 2011 were excluded based on the age criterion. Outpatients were defined as individuals who used WM or KM more than once in 2011, and 2,376 persons who did not use outpatient services or who used only dental or maternity care services were excluded. The final number of subjects was 9,753, as 554 who either did not or could not answer the questionnaire were excluded (Fig. 1).

Among the final subjects, KM users were defined as those who used KM more than once in 2011; the subjects included 1,870 individuals who used WM and KM jointly and 76 who used KM only. The 7,807 individuals classified as KM nonusers were those who used outpatient services other than dental or maternity clinic services more than once and did not use KM in 2011.

### Model

Andersen [16] suggested an initial behavioral model in which predisposing characteristics, enabling resources and needs influence the use of health services. He also noted that health beliefs (i.e., attitudes and values about health and health services) affect the use of health services as predisposing characteristics [16]. According to Andersen’s model, we included MD satisfaction with other health beliefs as independent variables, and we added predisposing characteristic variables, enabling resource variables, and needs variables as covariates that modify the use of KM.

### MD satisfaction

Initially, the survey questionnaire contained 7 indicators with a 4-point Likert scale regarding MD satisfaction; ‘trust in the MD’, ‘MD’s careful listening’, ‘MD’s sufficient explanation’, ‘MD’s consultation time’, ‘MD’s respect for patient’, ‘frequency of health care facility visits’ and ‘usefulness of screening’. We performed a factor analysis to examine the conceptual congruence of these 7 indicators, and, as a result, we excluded 2 indicators during the analysis; ‘frequency of health care facility visits’ and ‘usefulness of screening’ (Table 1). Then, we combined remaining 5 indicators into one factor. This factor showed good internal consistency, and Cronbach’s alpha was 0.8591. We additionally generated an “overall MD satisfaction” variable by using the mean value of the 5 indicators in this factor.

**Table 1:** Questionnaire on patients’ satisfaction with their medical doctors

1. Trust in the MD	Do you generally trust the MD you visited?
	① Strongly agree ② Somewhat agree ③ Somewhat disagree ④ Strongly disagree
2. MD’s careful listening	Did the MD you visited listen attentively?
	① Strongly agree ② Somewhat agree ③ Somewhat disagree ④ Strongly disagree
3. MD’s sufficient explanation	Did the MD you visited provided easy-to-understand medical explanations?
	① Strongly agree ② Somewhat agree ③ Somewhat disagree ④ Strongly disagree
4. MD’s consultation time	Did the MD you visited allocate enough consultation time to meet your needs?
	① Strongly agree ② Somewhat agree ③ Somewhat disagree ④ Strongly disagree
5. MD’s respect for the patient	Did the MD you visited respect what you said?
	① Strongly agree ② Somewhat agree ③ Somewhat disagree ④ Strongly disagree

### Covariates

In this study, the covariates comprised predisposing characteristics, enabling resources and needs that are known to have an effect on health care use in Andersen’s behavior model. The predisposing characteristic variables are age, gender, education and residential area. The residential areas listed in the questionnaire included 16 Korean cities and provinces, but we condensed these to 5 areas (Seoul metropolitan area, Chungcheong-Gangwon, Yeongnam, Honam, and Jeju) in consideration of the cultural traits and geographic proximity of the residential areas.

Enabling resource variables included marital status, work status, household income and residential characteristics; residential characteristics were divided into urban and rural. Need variables were composed of the number of patients with and without chronic diseases, hypertension, diabetes, arthritis, and neoplasms; a subjective health status assessment; and quality of life. Among these variables, the quality-of-life variable was calculated by scores on the EQ-5D profile adjusted by a South Korean time trade-off value [17]. Subjective health status was surveyed with a 5-point Likert scale; the higher the score, the more negative one’s perception of one’s health.

### Analysis

The chi-square test (categorical variables) and *t*-test (continuous variables) were used to examine differences in the use of KM according to MD satisfaction variables and covariates. Then, we entered covariates found to be significantly associated with CAM use in the univariate analysis into a multiple logistic regression model. The criterion for covariate entry was *p* = 0.2. However, although all variables of MD satisfaction were *p* >0.2, we included them in the analysis. We then sequentially added the variables for 1) MD satisfaction; 2) predisposing factor; 3) enabling factor; and 4) need factor to the unadjusted model.

Each logistic regression analysis that was conducted included a goodness-of-fit test and c-statistic. In addition, we measured the multi-collinearity between the variables. All analyses were performed using Stata/IC, version 12.1 (Stata Corp, College Station, TX). Hypothesis testing was conducted using an alpha level of 0.05.

## Results

Among the 9,753 study subjects, 1,946 (20.0 %) used KM. 1,946 (20.0 %). The KM users consisted of 1,351 women (23.6 %), 628 with an elementary school education (25.7 %), 898 with non-economic activities (21.9 %), 590 in the bottom 25 % of household income (24.0 %), 980 with more than three chronic diseases (28.6 %), 565 with hypertension (22.3 %), and 467 with arthritis (32.7 %). The KM users’ mean age was 55.6, which was lower than that of non-users. The users’ mean EQ-5D score was low (0.892), and their subjective health status was poor (2.836) (Table 2).

**Table 2:** Descriptive statistics and bivariate analysis for the demographics of users and nonusers of KM

		Nonusers		Users		*p*-value
		*N*	%	*N*	%	
Predisposing characteristic variables						
Gender	Male	3,423	85.19	595	14.81	<0.001
	Female	4,384	76.44	1,351	23.56	
Age (yrs)	mean ± SD	51.503 ± 16.207		55.608 ± 15.575		<0.001
Education	≤ Elementary school	1,815	74.29	628	25.71	<0.001
	≤ High school	3,495	80.34	855	19.66	
	> High school	2,497	84.36	463	15.64	
Region	Seoul metropolitan area	3,229	81.44	736	18.56	0.003
	Chungcheong-Gangwon	1,054	78.42	290	21.58	
	Yeongnam	2,342	79.69	597	20.31	
	Honam	976	77.40	285	22.60	
	Jeju	206	84.43	38	15.57	
Enabling resource variables						
Spouse	Yes	5,945	80.45	1,445	19.55	0.081
	No	1,862	78.80	501	21.20	
Work status	Not employed	3,208	78.13	898	21.87	<0.001
	Employed	2,880	82.07	629	17.93	
	Self-employed	1,719	80.40	419	19.60	
Household annual income low level (%)	<25	1,869	76.01	590	23.99	<0.001
	<50	1,955	80.69	468	19.31	
	<75	2,012	82.70	421	17.30	
	≥75	1,971	80.84	467	19.16	
Regional characteristic	Rural	3,390	80.09	843	19.91	0.935
	Urban	4,417	80.02	1,103	19.98	
Needs variables						
Chronic disease	No	2,485	86.62	384	13.38	<0.001
	<3	2,878	83.18	582	16.82	
	≥3	2,444	71.38	980	28.62	
Hypertension	No	5,837	80.87	1,381	19.13	0.001
	Yes	1,970	77.71	565	22.29	
Diabetes	No	7,053	80.28	1,732	19.72	0.077
	Yes	754	77.89	214	22.11	
Arthritis	No	6,846	82.23	1,479	17.77	<0.001
	Yes	961	67.30	467	32.70	
Neoplasm	No	7,363	80.36	1,800	19.64	0.003
	Yes	444	75.25	146	24.75	
EQ-5D	mean ± SD	0.910 ± 0.088		0.892 ± 0.097		<0.001
Subjective health condition	mean ± SD	2.680 ± 0.858		2.836 ± 0.855		<0.001

When we did not control for variables, there was no significant difference between the MD satisfaction of KM users and that of non-users in any of the 5 indicators. In addition, there was no statistically significant difference in the overall MD satisfaction derived from the mean of the 5 indicators (Table 3).

**Table 3:** Satisfaction with MDs for users and nonusers of KM

		Nonusers		Users		*p*-value
		*N*	%	*N*	%	
Trust in the MD	Dissatisfaction	755	81.98	166	18.02	0.124
	Satisfaction	7,052	79.85	1,780	20.15	
MD’s careful listening	Dissatisfaction	731	81.86	162	18.14	0.155
	Satisfaction	7,076	79.86	1,784	20.14	
MD’s sufficient explanation	Dissatisfaction	852	80.53	206	19.47	0.678
	Satisfaction	6,955	79.99	1,740	20.01	
MD’s consultation time	Dissatisfaction	2,095	79.00	557	21.00	0.113
	Satisfaction	5,712	80.44	1,389	19.56	
MD’s respect for the patient	Dissatisfaction	931	79.10	246	20.90	0.386
	Satisfaction	6,876	80.18	1,700	19.82	
Overall MD satisfaction	mean ± SD	2.960 ± 0.401		2.956 ± 0.380		0.670

As a result of the logistic regression, however, when controlling for predisposing characteristic variables, enabling resource variables and needs variables, the more people were dissatisfied with their doctor, the more they used KM (OR = 0.87). Specifically, the more a patient was dissatisfied with the “consultation time of the MD”, the more they used KM (OR = 0.82), whereas “trust in the MD”, “MD’s careful listening”, “MD’s sufficient explanation” and “MD’s respect for the patient” did not show a statistically significant influence on the use of KM (Table 4).

**Table 4:** Results from multivariate analysis for users and nonusers of KM

	Unadjusted	+Predisposing variables	+Enabling variables	+Need variables
Trust in the MD	1.18 (0.96–1.44)	1.13 (0.92–1.34)	1.13 (0.92–1.38)	1.12 (0.91–1.38)
MD’s careful listening	1.23 (0.98–1.56)	1.23 (0.98–1.56)	1.23 (0.97–1.56)	1.18 (0.93–1.50)
MD’s sufficient explanation	1.04 (0.85–1.27)	1.04 (0.85–1.27)	1.03 (0.84–1.27)	1.06 (0.86–1.30)
MD’s consultation time	0.88^*^(0.77–1.00)	0.82^**^(0.71–0.93)	0.82^**^(0.72–0.93)	0.82^**^(0.72–0.94)
MD’s respect for the patient	0.86 (0.71–1.04)	0.84 (0.69–1.03)	0.84 (0.69–1.03)	0.85 (0.70–1.04)
Log likelihood	−4868.1342	−4747.1273	−4743.7057	−4651.1695
Goodness-of-fit test (*p*-value)	0.5478	0.1363	0.5438	0.4848
c-Statistic	0.5187	0.6170	0.6189	0.6513
Mean VIF	1.56	1.55	1.62	1.63
Overall MD satisfaction	0.97 (0.86–1.10)	0.88^*^ (0.77–1.00)	0.88^*^ (0.77–1.00)	0.87^*^ (0.76–0.99)
Log likelihood	−4873.956	−4753.6	−4749.9115	−4655.9882
Goodness-of-fit test (*p*-value)	0.0223	0.4538	0.4323	0.4997
c-Statistic	0.5136	0.6143	0.6161	0.6491
Mean VIF	-	1.48	1.60	1.61

(unit: OR, 95 % Conf. Interval)

*: *p* <0.05 **: *p* <0.01

## Discussion

There have been many previous studies on whether MD satisfaction influences the use of CAM, but the results varied. Downer [11] and Sirois [6] noted that CAM users showed a great deal of dissatisfaction with conventional medicine, whereas Eisenberg [18] and Astin [8] noted that the use of CAM was not influenced by dissatisfaction with conventional medicine or MDs. The studies noting that the use of CAM was associated with dissatisfaction with conventional medicine suggested that people think conventional medicine alone cannot help fight disease and is not helpful [6, 10, 11], and the studies indicated that this distrust of treatment effectiveness was more important than the doctor-patient relationship or the patient’s beliefs regarding health and medicine [6, 11]. The British Medical Association’s Board of Science and Education [19] also noted that the most significant reason for the success of CAM has been the failure of modern science and conventional medicine to treat incurable diseases.

It was difficult to verify directly how much the treatment effectiveness of conventional medicine related to dissatisfaction with MDs because we did not include perception of treatment effectiveness of conventional medicine as an explanatory variable. However, among the 5 indicators used for variables, “trust in the MD” could be considered the most relevant to treatment effectiveness, so we analyzed this variable. The level of “trust in the MD” did not affect the use of KM. Rather, KM users had a tendency to show higher trust in their doctor than KM non-users, although the difference was not statistically significant. This appears to be in agreement with the results of a previous study [18] indicating that most CAM users believe it is better to use both conventional medicine and CAM rather than to discard the treatments of conventional medicine. In other words, KM users use KM not as a substitute for conventional medicine but as a complementary and additional type of medicine. Choi [20] also reported that KM has a complementary relationship with conventional medicine.

We consider that “trust in the MD” is the most relevant indicator regarding the treatment effectiveness of conventional medicine, but Paltiel [9] explained that “trust in the MD” is relevant to authority rather than effectiveness. He also suggested that the reason for incomplete trust in the doctor is that patients try not to accept patriarchal and authoritative conventional medicine. Thus, we could allow that “trust in the MD” combined both trust in treatment effectiveness and factors such as intimacy, kindness, and faithfulness.

The results of our analysis revealed that the biggest reason for dissatisfaction with MDs was the lack of consultation time. Many international studies have reported that CAM doctors spend more time with their patients than do traditional MDs [18, 21–23]. In Korea, similar results were reported showing that an MD’s average consultation time per patient was 2 min and 34 sec at clinics, whereas a KM doctor’s consultation time was 3 min and 19 sec at clinics [24]. In Korea, the number of MDs was 2.1 per 1,000 people, the lowest among OECD member countries (Avg. 3.2) [25]. The number of outpatient visits (per capita) was 14.3, which was the second highest among OECD countries (Avg. 6.9) [25]. This situation forces MDs to see more patients during a given time to gain more economic benefit; therefore, we could consider that complaints about insufficient consultation time make the use of KM more attractive.

This study reconfirms the results of a previous study [18] showing that patients used CAM not only to alleviate their dissatisfaction with conventional medicine but also to seek, explore, and experience benefits from both conventional and CAM therapies. Our study results were in contrast to those of another study [11] that showed that patients with severe disease used CAM to gratify psychological needs for medical service; our results showed that patients with severe disease were highly affected by psychological needs such as a doctor’s respect, which is related to KM use. These findings indicate that patients who use KM do not abandon conventional medicine but demand more responsibility from the medical community as a broader distributor of health and medical services, and conventional medicine and CAM should be integrative partners in the health and medical fields to help improve patients’ psychological health [18].

Our study has some limitations. First, we could not identify a causal relationship between the use of KM and variables known to be related to the use of KM by cross-sectional analysis. Second, the health beliefs influencing the use of health services include attitudes, values, and knowledge [16] about health and health services, but we only included variables regarding the doctor-patient relationship due to the limitations of the Korea Health Panel data. Finally, the term KM doctor is legally separate from MD. Some people, however, could not distinguish KM doctors from MDs because a KM doctor’s legal status is very similar to a WM doctor’s. Some respondents in the Korea Health Panel survey might have had similar confusion when they answered the questionnaire; this might have caused information bias.

Despite the limitations mentioned above, this study is the first attempt to address the relationship between MD satisfaction and the use of KM and provides reliable results by adjusting the variables of respondents’ socio-demographic characteristics, which were categorized with Andersen’s behavior model.

## Conclusions

Patients who were dissatisfied with their MD were more likely to use KM; the main indicator affecting MD dissatisfaction was the relatively short time of MD consultations. This could be one reason why KM plays a complementary role with conventional medicine in Korea.

## Abbreviations

KM: Traditional Korean medicine
CAM: Complementary and alternative medicine
MD: Western medicine doctor
WM: Western medicine
KHP: Korea health panel
OR: Odds ratio
